# Lower extremity amputations (LEAs) in a tertiary hospital in Togo: a retrospective analysis of clinical, biological, radiological, and therapeutic aspects

**DOI:** 10.1186/s13018-023-03628-5

**Published:** 2023-03-02

**Authors:** T. E. Kouevi-Koko, K. S. Amouzou, A. Sogan, S. Apeti, Y. E. L. Dakey, A. Abalo

**Affiliations:** 1 Burn and Wound Healing Unit, Faculté des Sciences de la Santé, University of Lomé, Lomé, Togo; 2 Department of General Surgery, Faculté des Sciences de la Santé, University of Lomé, Lomé, Togo; 3 Laboratory of Human Anatomy, Faculté des Sciences de la Santé, University of Lomé, Lomé, Togo; 4 Department of Geriatrics, Faculté des Sciences de la Santé, University of Lomé, Lomé, Togo; 5 Traumatology-Orthopaedics Department, Faculté des Sciences de la Santé, University of Lomé, Lomé, Togo; 6 General Surgery, Faculté des Sciences de la Santé, University of Lomé, Lomé, Togo; 7 Sylvanus Olympio Teaching Hospital, Lomé, Togo

**Keywords:** Amputation, Lower extremity, Diabetes mellitus, Diabetic foot, Togo

## Abstract

**Background:**

We analysed the clinical, biological, radiological profiles, and therapeutic patterns of the patients who underwent a surgical lower extremity amputation (LEA) in Togo from 2010 to 2020.

**Methods:**

Retrospective analysis of clinical files of adult patients who underwent an LEA at a single centre (Sylvanus Olympio Teaching Hospital) from 1st January 2010 to 31st December 2020. Data were analysed by CDC Epi Info Version 7 and Microsoft Office Excel 2013 software.

**Results:**

We included 245 cases. The mean age was 59.62 years (15.22 SD) (range: 15–90 years). The sex ratio was 1.99. The medical history of diabetes mellitus (DM) was found in 143/222 (64.41%) files. The amputation level found in 241/245 (98.37%) files was the leg in 133/241 (55.19%) patients, the knee in 14/241 (5.81%), the thigh in 83/241 (34.44%), and the foot in 11/241 (4.56%). The 143 patients with DM who underwent LEA had infectious and vascular diseases. Patients with previous LEAs were more likely to have the same limb affected than the contralateral one. The odds of trauma as an indication for LEA were twice as high in patients younger than 65 years compared to the older (OR = 2.095, 95% CI = 1.050–4.183). The mortality rate after LEA was 17/238 (7.14%). There was no significant difference between age, sex, presence or absence of DM, and early postoperative complications (*P* = 0.77; 0.96; 0.97). The mean duration of hospitalization marked in 241/245 (98.37%) files was 36.30 (1–278) days (36.20 SD). Patients with LEAs due to trauma had a significantly longer hospital admission than those with non-traumatic indications, F (3,237) = 5.505, *P* = 0.001.

**Conclusions:**

Compared to previous decades, from 2010 to 2020, the average incidence of LEAs for all causes at Sylvanus Olympio Teaching Hospital (Lomé, Togo) decreased while the percentage of patients with DM who underwent LEAs increased. This setting imposes a multidisciplinary approach and information campaigns to prevent DM, cardiovascular diseases, and  relative complications.

## Background

The term “amputation” derives from the Latin word “amputare” (“to excise” or “to cut out”), which means the removal of part or all of a body part delimited by the skin. Lower extremity amputation (LEA) consists of removing lower limbs by accident or surgery [1]. LEA is a severely disabling condition that imposes variable physical and psychological challenges as it entails lifestyle changes, self-concept alterations, impairments in physical functioning, and sensory effects [2–5]. LEA can occur as complications of trauma or underlying diseases, such as infections, tumours, cardiovascular or vascular diseases, especially diabetes mellitus (DM), and neglected congenital conditions [6, 7]. The incidence of LEA varied from 8.8 per 100.000 people in the Netherlands in 2004 to 92.5 per 100.000 in Ireland in 2009 [8, 9]. However, in Western countries, LEA has been recently reported as declining [10–12]. In Africa, the epidemiological studies about LEA for all the causes are limited. Although the predominant aetiologies of LEAs in the continent have been reported as trauma, infections of wounds, and leg ulcers [13, 14], recent data have referred most to DM. The incidence of DM-driven LEAs in Ghana increased from 0.6 in 2010 to 10.9 in 2015 [15], in Malawi, the rate was 10.6% in 2008–2016 [16] and, in South Africa, 53.7% in 2013–2018 [17]. In Nigeria, the LEA rate in patients with diabetic foot ulcer disease was 35.4% during 2016–2017 [18]. In the review of Abbas et al. (1960–2020), the amputation rate in patients with diabetic foot ranged from 3 to 61% [19]. In Togo, Abalo et al. reported a predominance of vascular causes of LEA from January 2000 to December 2004; among these vascular disorders, DM accounted for 48.2% [20]. In 2017, Amouzou et al. showed that all the LEAs due to leg ulcers were complications of diabetic foot [21].

The present study has described the clinical, biological, radiological profiles and therapeutic patterns of patients who underwent LEA in Togo from 2010 to 2020. In particular, we have analysed the characteristics of patients with DM who had LEA at Sylvanus Olympio Teaching Hospital (SOTH), which is the only one of the three tertiary hospitals in Togo equipped with a Wound Healing Unit.

## Methods

This retrospective study was based on data retrieved from clinical files of adult (> 15-year-old) patients admitted and treated in the Wound Healing Unit and Traumatology-Orthopaedics department of Sylvanus Olympio Teaching Hospital (SOTH), in Lomé, the capital of Togo, from 1st January 2010 to 31st December 2020. Togo is a country in West Africa of 56,600 km^2^, with a population of 8,849,000 in 2022, among which 5% were above 60 years. Togo was the first country to eradicate four tropical diseases (dracuncunculiasis, lymphatic filariasis, human African trypanosomiasis, and trachoma) [22]. Togo’s health system is relatively well-equipped in terms of infrastructures, and 70.9% of the population has access to facilities. However, geographical, economic, and social disparities regarding the supply and accessibility of essential health care persist. Analysis of the distribution of human resources for health indicates that most of the health workforce is in the capital; rural areas are disadvantaged in this respect [23]. The health system in Togo is pyramidal with three levels: central, intermediate, and peripheral. The base represents the peripheral level with district hospitals, maternal and infant protection centres (PMI), and peripheral care units (USP). The middle of the pyramid represents the intermediate or regional level and corresponds to six health regions with six regional hospitals. Finally, the top of the pyramid, called the central or national level, encompasses the office of the Minister of Health, its national directions, and the three teaching hospitals. Two of the three teaching hospitals are in the capital, among which is SOTH, and one in the North of the country in Kara.

The patients admitted at SOTH enrolled in the current study came from Lomé and its surrounding areas.

This study was conducted according to the World Medical Association’s Declaration of Helsinki (1964, version 2013) [24], Good Clinical Practice, and approved by the medical. Institutional Board of SOTH. The drafting of the manuscript complied with the STROBE guidelines [25].

From the clinical files, we retrieved the following data:

- *Socio-demographic parameters* Age (“aged” patients were those 65-year-old and older), sex, job;
- *Past medical conditions and chronic diseases* DM, hypertension, sickle cell disease;
- *Charlson Comorbidity Index* (CCI) was calculated from the files, and distributed in 4 groups: 0 (no comorbidity), [1–4], ≥ 5 [26, 27];
- *Clinical characteristics* Ischaemic or wet gangrene, tumour, crush syndrome;
- *Biological parameters* Haemoglobin rate, white cells count;
- *Radiological findings* Vascular lower limb ultrasonography;
- *Histological nature of the amputated part*;
- *Therapeutic aspects and operative records* Type of anaesthesia, side of amputation, amputation level (minor amputation: toe or part of the foot; major amputation: above and below knee), duration between indication and surgery, duration of hospital admission;
- *Operative course* Immediate and early postoperative complications (present or absent), which included death, psychological troubles, wound infections (surgical site infections), the persistence of ischaemia, and disorders on the contralateral limb. The immediate complications were defined as those that happened in the first 24 h after surgery and the early those that happened from the first 24 h after surgery to the supposed date wound healing;
- *Clavien–Dindo classification modified for foot and ankle surgery* Ia (likely negligible), Ib (likely minimal), IIa (likely minimal), IIb (likely significant), IIIa (likely minimal), IIb (likely significant), IV, V (serious) [28, 29].

We included clinical files of all adult patients who underwent an LEA at SOTH, of both sexes. We excluded missing or incomplete clinical files. We defined the incomplete files as those with more than half of the above parameters missing.

We performed a descriptive statistical analysis of the data with CDC Epi Info Version 7.2.0.1 and Microsoft Office Excel 2013 software and provided the Chi-square value, degree of freedom, Fisher test, Odds Ratio (OR), confidence interval, and cell value P, if necessary. The cell values under 5% were considered significant.

We used Microsoft Office Excel 2013 software for drafting tables and figures. We used the mean value to measure the age of the patients, rate of haemoglobin, hospital stay, and duration from indication to LEA.

## Results

### General data

**Fig. 1:**
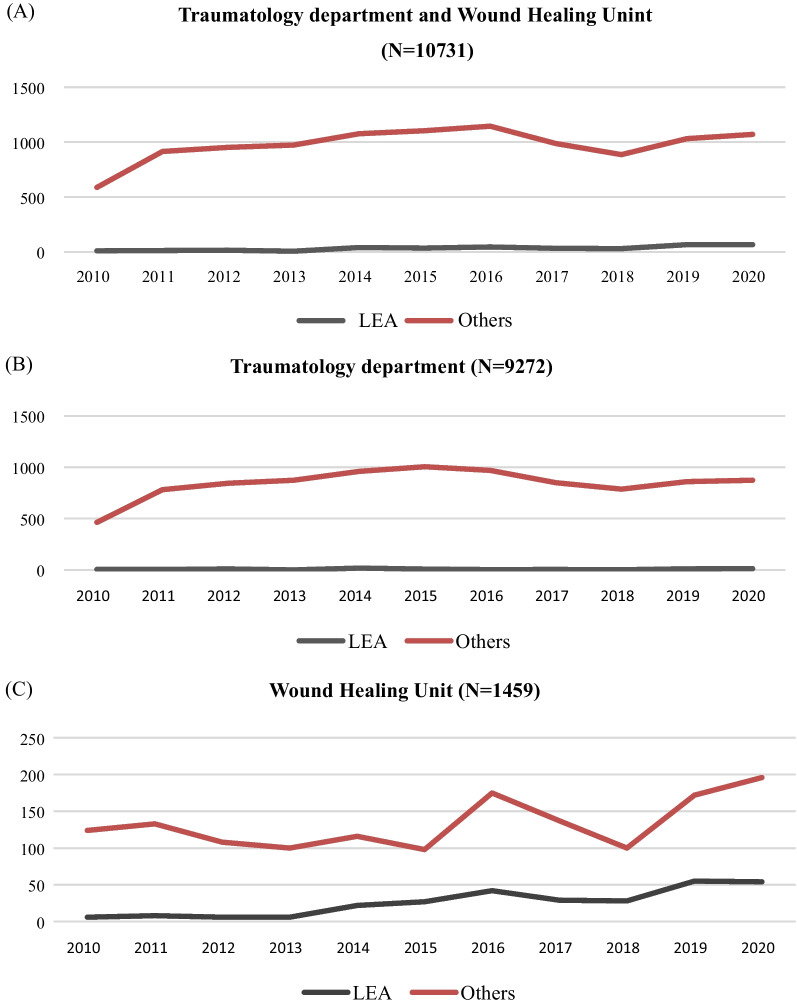
Distribution of patients admitted in Traumatology department and Wound Healing Unit (**A**), in Traumatology department (**B**), and Wound Healing Unit (**C**) for LEA or others by year

In the study period of 11 years (2010–2020), a total of 10,731 patients were admitted to the Traumatology-Orthopaedics department and Wound Healing Unit of Sylvanus Olympio Teaching Hospital. LEA was conducted in 363/10731 (3.38%) patients, and the average number of LEAs per year was 33. Figure 1 displays the distribution of all the patients admitted to both the Traumatology-Orthopaedics department and Wound Healing Unit (Fig. 1A), to the only Traumatology-Orthopaedics department (Fig. 1B), and the only Wound Healing Unit (Fig. 1C).

**Fig. 2:**
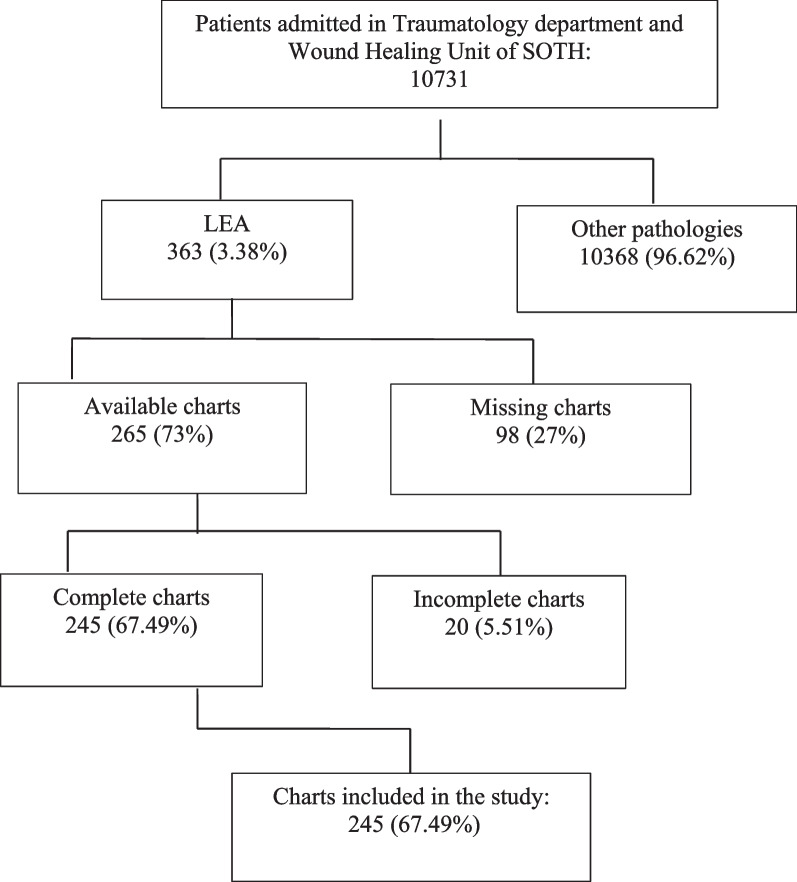
Flow diagram of the participants throughout the study phases

The clinical files of 98/363 (27%) patients operated on for LEA were missing. Of the 363 clinical files, 20 (5.51%) had incomplete information, and 245 (67.49%) contained enough information to be included in this study. Figure 2 shows the flow diagram of the participants throughout the study phases.

### Demographic data

**Fig. 3:**
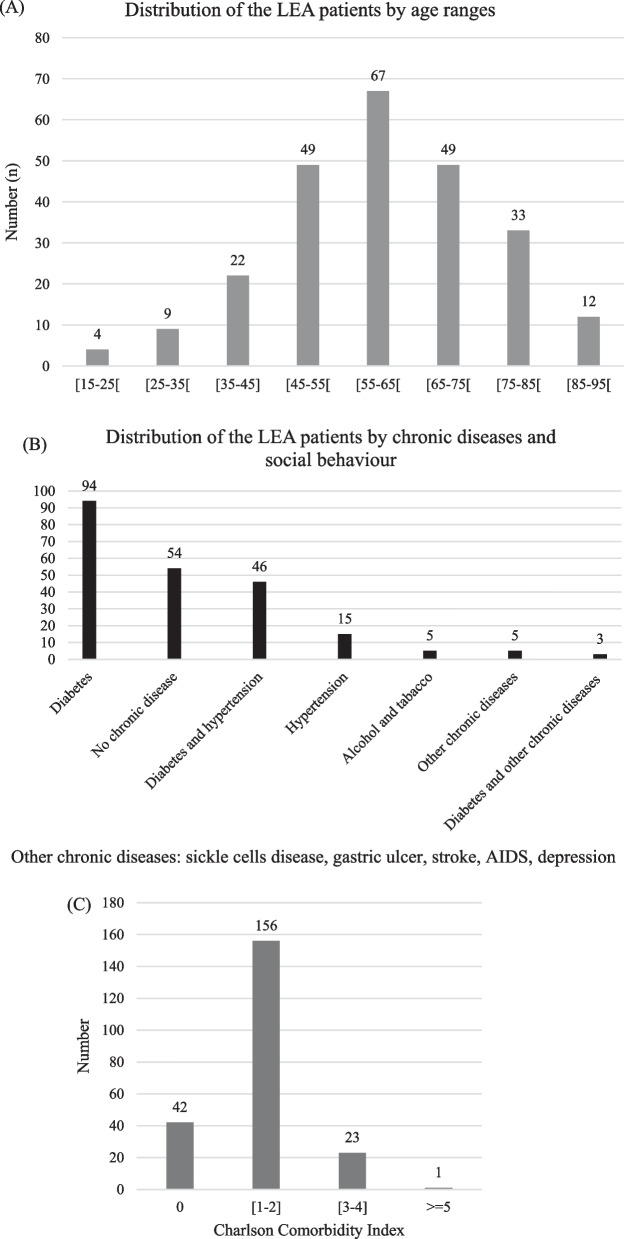
Distribution of the LEA patients by age ranges (**A**), chronic diseases and social behaviour (**B**) (N = 245), and comorbidity index (**C**) (N = 222)

The mean age of the 245 patients who underwent LEA was 59.62 years (15.22 SD) (range: 15–90 years). Figure 3A displays the age distribution; patients more than 65-year-old accounted for 94/245 (38.37%).

Among the various patients’ professions, tailoring was the most frequent (22.86%, *P* = 0.023) (Table 1).

**Table 1:** Distribution of patients according to their profession

	n	%
Tailor	56	22.86
Trader	20	8.16
Carpenter	15	6.12
Welder	12	4.90
Teacher	12	4.90
Driver	12	4.90
Unemployed	12	4.90
Housewife	12	4.90
Retired civil servant	12	4.90
Farmer	8	3.27
Retired teacher	8	3.27
Traditional priest	8	3.27
Painter	8	3.27
Ambulance driver	5	2.04
Cooker	5	2.04
Docker	4	1.63
Macon learner	4	1.63
Custom inspector	4	1.63
Retired farmer	4	1.63
Macon	4	1.63
Retired soldier	4	1.63
Manual labourer	4	1.63
Electrician	4	1.63
Policeman	4	1.63
Student	4	1.63
Total	245	100.00

The sex ratio of our patient population was 1.99. There was a statistically significant difference between male (163, 66.53%) and female patients (82, 33.47%) (*P* < 0.001).

### Medical and social history data

In the medical history of the 245 patients included in this study, 26 (10.61%) had a previous LEA. The previous LEAs of the 26 patients were minor (50%) and major (50%) amputations.

Of the 245 clinical files, the past medical conditions were missing in 23 (9.39%) clinical files and present in 222 (90.61%). Of the 222 clinical files with data about past medical conditions, 143 patients had DM. Of them, 94 (42.34%) had DM alone, and 49 (22.07%) had DM associated with other comorbidities (Fig. 3B). Two hundred and four out of 222 (91.90%) patients had comorbidity for more than one year, 6 (2.70%) between 6 months and one year, and 12 (5.40%) for less than 6 months. The Charlson Comorbidly Index (CCI) was 0 in 18.92%, and below 2 in 89.19% (Fig. 3C).

Twenty-six out of 222 (11.71) patients had previous LEAs; of them, 20 (76.92%) were diabetic, and 6 (23.08%) were non-diabetic.

### Clinical, biological, radiological data and LEA causes

**Fig. 4:**
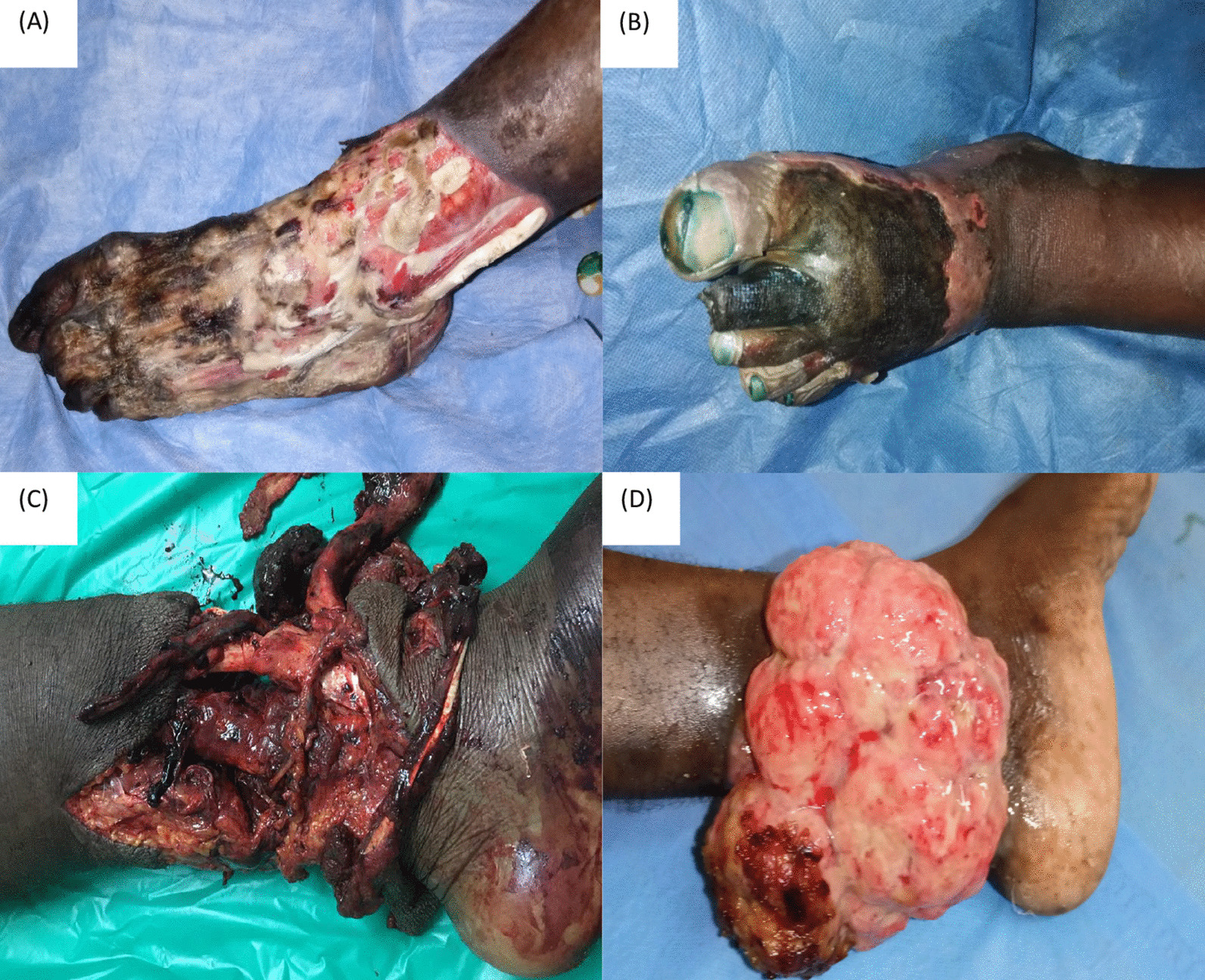
Clinical aspects of patients with LEA. **A**–**B** Foot gangrene,** C** Crush in the leg, **D** Ankle tumor

Gangrene was the clinical presentation of the limb at hospitalization in 212/245 (86.53%) patients. Other clinical presentations were crush syndrome in 23/245 (9.39%), bone tumour in 5/245 (2.04%), osteomyelitis in 2/245 (0.82%), chronic ulcer in 2/245 (0.82%), and necrotizing fasciitis in 1/245 (0.40%) cases. Figure 4 shows the clinical aspects of some patients who underwent LEA. We found the data about the type of gangrene in 194/212 (91.51%) clinical files; of them, 71 (36.60%) were ischaemic gangrene, and 123 (63.40%) were wet. Of the 123 patients with wet gangrene, 16 (13.01%) were with gas gangrene.

The 26 patients with previous amputations were more likely to have the same limb affected than the contralateral limb. The ipsilateral limb was amputated in 57.7% of patients compared to 42.5% of those with a contralateral limb (*P* < 0.001). The contralateral limb was amputated in 23.08% of patients after a minor amputation and in 53.85% after a major one.

Previous amputation was less common in patients with traumatic indications (5.9%) than in those with non-traumatic (11.9%), yet this difference did not reach statistical significance ((OR = 0.465, 95% CI = 0.134–1.614), *P* = 0.308).

A complete blood count was registered in 235/245 (95.92%) clinical files. Patients with hyperleukocytosis accounted for 153/235 (65.11%). The count of the white blood cells was in the normal range for 80/235 (34.04%) patients; 2/235 (0.85%) patients had leukopenia. The average count of white blood cells was 15,646 elements/mm^3^ (9.25DS) (range: 3200–44,700). The rate of white cell anomalies (leukopenia and hyperleukocytosis) was significantly higher in patients with DM compared to those without DM (98% vs. 43%, *P* = 0.01). Patients with infection had the highest white blood cell counts, while those with tumours had the lowest haemoglobin (Table 2). The mean rate of haemoglobin was 9.33 g/dl (2.38DS) (range: 2.5–17); 36 out of 235 patients had a haemoglobin rate under 7 g/dl.

**Table 2:** Association between amputations’ aetiology and full blood count results of the patients

	Average results	Statistical test	*P*
*Average WBC/000*mm^3^* (SD)*			
Infection	18.08 (9.52)	F (3,231) = 10.802	< 0.001
Tumour	14.26 (7.87)		
Vascular	13.70 (8.39)		
Trauma	8.02 (3.67)		
*Average HB g/dl (SD)*			
Tumour	7.72 (5.52)	F (3,237) = 2.154	0.094
Infection	9.17 (2.19)		
Vascular	9.44 (2.37)		
Trauma	10.22 (2.43)		

Doppler-ultrasound was performed in 91/245 (37.14%) patients; 18/91 (19.78%) patients were in the normal range, 42/91 (46.15%) displayed arterial abnormality (peripheral artery disease), 6/91 (6.59%) had venous disorders (superficial insufficiency or deep venous thrombosis), and 25/91 (27.48%) a combined artery and venous disorders.

**Fig. 5:**
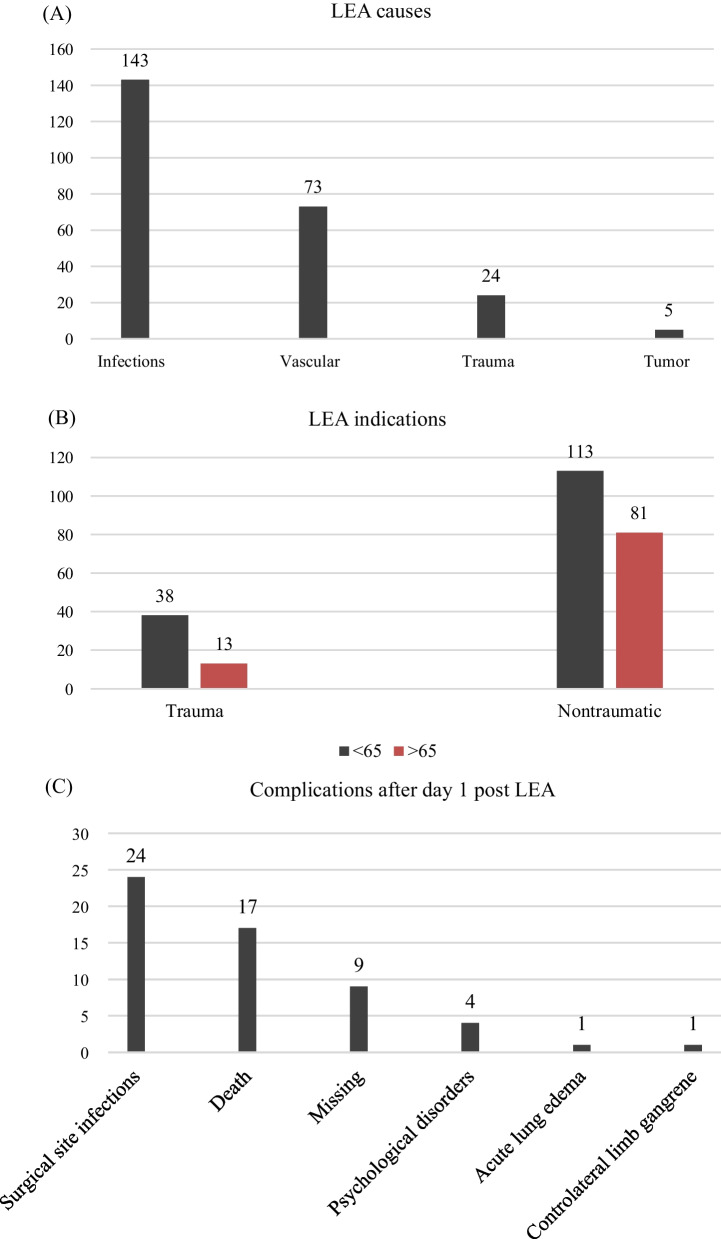

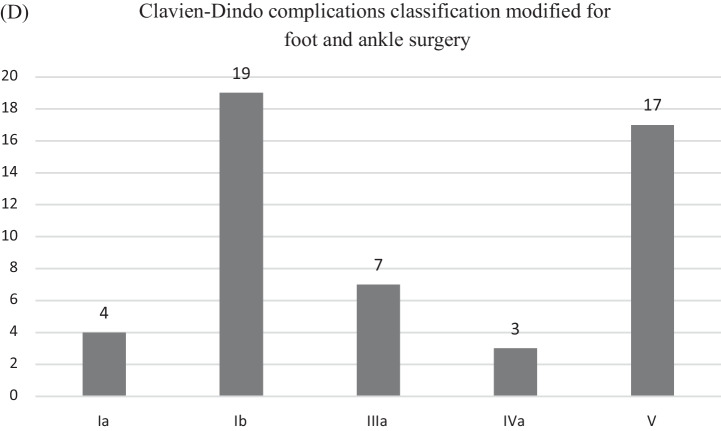
Numerical distribution of patients by causes of LEA (N = 245) (**A**), number of traumatic or nontraumatic LEA according to the range of patient’s age (< 65 years or > 65 years) (N = 245) (**B**), numerical distribution of the early complications (N = 238) (**C**), and the distribution of complications according to the modified Clavien–Dindo classification for foot and ankle surgery (**D**)

The causes of LEA were infections in 143/245 (58.37%) cases and bone or skin tumours in 5/245 (2.04%) (Fig. 5A). No data about histology types of the tumour was documented. The 143 patients with DM who underwent LEA were involved in infectious and vascular aetiologies.

When we categorized age into less or more than 65 years, there was a significant association between age and the cause of LEA. Patients younger than 65-year-old had significantly more traumatic indications for LEA than those older (*P* = 0.023). The odds of trauma as an indication for LEA were twice as high in those younger than 65 years compared to the older (OR = 2.095, 95% CI = 1.050–4.183). Figure 5B shows the relationship between traumatic indications for LEA and the patient’s age.

### Therapeutic data

Spinal anaesthesia was administered in 242/245 (98.78%) patients and general anaesthesia to 3/245 (1.22%). The side of the LEA was reported in 239/245 (97.55%) clinical files and missing in 6/245 (2.45%). LEA was left in 116/239 (48.54%) patients and right in 123/239 (51.46%). We found data about the amputation level in 241/245 (98.37%) clinical files: leg in 133/241 (55.19%) patients, knee in 14/241 (5.81%), thigh in 83/241 (34.44%), and foot in 11/241 (4.56%). There was no significant association between the aetiology of the LEA and the amputation level (*P* = 0.052) (Table 3). The amputation level below the knee was significantly higher in patients with DM than in those without (96% vs. 39%, *P* = 0.01).

**Table 3:** Association between amputations’ aetiology and amputation level

	Numbers (%)	χ^2^ test	*P*
	n (%)		
*Infection*			
AKA	48 (19.59)	χ^2^ (12,245) = 22.814	0.052
BKA	81 (33.06)		
Foot	4 (1.63)		
Through knee	8 (3.27)		
Missing values	2 (0.82)		
*Trauma*			
AKA	6 (2.45)		
BKA	14 (5.71)		
Foot	3 (1.22)		
Missing values	1 (0.41)		
*Tumour*			
AKA	3 (1.22)		
BKA	1 (0.41)		
Missing values	1 (0.41)		
*Vascular*			
AKA	26 (10.61)		
BKA	37 (15.10)		
Foot	4 (1.63)		
Through knee	6 (2.45)		
Total	245 (100.00)		

The mean duration from the indication of amputation to the surgery found in 239/245 (97.55%) clinical files was 6.75 (1–78) days (11.59 SD).

In the 237 out of 245 (96.73%) clinical files with recorded postoperative course, there was no immediate postoperative complications; there was a postoperative complication in 3/237 (1.27%); specifically, two patients had a stroke after the surgery, and one had a perioperative shock that raised favourably on medical treatment. Data regarding early postoperative complications were available in 238/245 (97.14%) clinical files. Complications were present in 56/238 (23.53%); the type of complications was reported in 47/56 (83.92%) clinical files and missing in 9/56 (16.08%) (Fig. 5C). According to Clavien–Dindo classification modified for foot and ankle surgery, the complications were likely minimal in 60%, and serious (life-threatening or involved central nervous system) in 40% (Fig. 5D).

Early postoperative complications did not significantly differ according to age (*P* = 0.77), sex (*P* = 0.96), level of amputation (*P* = 0.05), and presence or absence of DM (*P* = 0.97) (Table 4).

**Table 4:** Distribution of early postoperative complications in the follow-up according to age, sex, presence of diabetes, and level of amputation (N = 245)

	Early postoperative complications							
	Present		Absent		Total		Chi-2	*P*
*Other patients*								
Row %	111	75.51	36	24.49	147	100.00	0.0822	0.77
Col %		60.99		64.29		61.76		
*Aged patients*								
Row %	71	78.02	20	21.98	91	100.00		
Col %		39.01		35.71		38.24		
*Total*								
Row %	182	76.47	56	23.53	238	100.00		
Col %		100.00		100.00		100.00		
*Men*							0.0023	0.96
Row %	123	76.88	37	23.13	160	100.00		
Col %		67.58		66.07		67.23		
*Women*								
Row %	59	75.64	19	24.36	78	100.00		
Col %		32.42		33.93		32.77		
*Total*								
Row %	182	76.47	56	23.53	238	100.00		
Col %		100.00		100.00		100.00		
*Below knee*							3.6348	0.05
Row %	114	80.85	27	19.15	141	100.00		
Col %		63.69		48.21		60.00		
*Above knee*								
Row %	65	69.15	29	30.85	94	100.00		
Col %		36.31		51.79		40.00		
*Total*								
Row %	179	76.17	56	23.83	235	100.00		
Col %		100.00		100.00		100.00		
*Diabetes mellitus*							0.0009	0.97
Row %	105	75.54	34	24.46	139	100.00		
Col %		64.42		62.96		64.06		
*No diabetes*								
Row %	58	74.36	20	25.64	78	100.00		
Col %		35.58		37.04		35.94		
*Total*								
Row %	163	75.12	54	24.88	217	100.00		
Col %		100.00		100.00		100.00		

The mortality rate after LEA was 17/238 (7.14%). The mean hospital stay was marked in 241/245 (98.37%) clinical files and consisted of 36.30 (1–278) days (36.20 SD). The average hospital duration was 34.27 (27.86 SD) days for infections, 19.60 (20.62SD) for tumour, 32.44 (25.05 SD) for vascular aetiologies, and 63.25 (78.02 SD) for traumatic aetiologies. Patients with LEAs due to trauma had significantly longer hospital admission than those with non-traumatic indications (F (3,237) = 5.505, *P* = 0.001).

## Discussion

Our study has provided the updated pattern of LEA in Togo based on the data collected between 2010 and 2020 at the country’s main hospital (SOTH). The results have shown an overall improvement, but the causes of LEA and the socio-demographic features of the patients changed compared with the previous decades. However, the present study had an incomplete series of clinical files with data available for analyses.

Considering the increase of the population of Togo from 5.01 million in 2000, 6.57 million in 2010 to 8.44 million in 2020 [20, 30], the average number of LEA per year of 33 LEA found in our study has decreased compared to that previously reported of 32 by Abalo et al. in 2004. Therefore, in the last decades, the prevention of LEA in our country improved. However, the most remarkable increase in patients who underwent LEA occurred in the Wound Healing Unit of SOTH (Fig. 1C), especially from 2013 to 2020. This increase can be due to the more conservatory management of open injuries of lower limbs conducted in the Traumatology-orthopaedic department by the Plastic surgery team, which reduced the rate of traumatic LEAs. Although the Togolese ortho-plastic team was young, the protocol for the reconstruction of severe open fractures as in international guidelines was the radical “ortho-plastic” treatment with radical debridement, skeletal stabilization and soft tissue coverage [31]. Moreover, the first microvascular free gracilis microvascular reconstruction in April 2018 introduced microsurgery in Togo [32]. This intervention provided a relevant improvement and started a new era in the management of open injuries of lower limbs in our country.

Of the 363 patients who underwent LEA at SOTH during the study period (2010–2020), we found 245 folders available for analysis. Therefore, the missed data represent a bias of the present study given the considerable number of absent or incomplete clinical files. Moreover, the incomplete series of sociodemographic or clinical characteristics of inpatient cases, such as marital status, address, religion, ethnic group, residual limb length, or phantom limb sensation, prevented us from assessing a possible association between these characteristics and LEA and reduced the range of feasible statistical tests. However, the information obtained from the complete clinical files accounted for relevant features of the LEA in our setting.

The mean age of our patients who underwent LEA was 59.6 years. Ouchemi et al. in Tchad found a mean age of 47.0 years in 2016; Abalo et al. in Togo reported a mean age of 43.1 years in 2011, Kouassi et al. in Ivory Coast reported a mean age above 40 years in 2016; while Tobome et al. in Benin found a mean age of 37.4 years in 2015 [13, 14, 20, 33]. The mean age of our patients higher than those reported in the literature could be related to the high incidence of underlying cardiovascular disorders.

The sex ratio of our patient population was 1.99, with a statistically significant difference between female and male. Various authors reported the same male predominance in worldwide literature [1, 13, 20, 33, 34]. The greater tendency to inobservance and involvement in the trauma of men, which led to complications, could explain the higher proportion of LEA due to traumatic causes in men than women [35].

Concerning the occupation of the patients, although the percentage of tailors is nearly 23% of the professions, we have not identified any clear association between work activities and LEA.

In our series, the contralateral limb was more involved in LEA after major amputation than the minor. Some authors have reported similar data in the literature. Glaser et al. in the US found contralateral major amputation in 11.5% of patients and in 8.4% after a minor amputation at 5 years [36]. Huseynova et al. in Canada reported an incidence of contralateral major amputation of 4.8 and 2.2 after a minor amputation [37].

In our series, DM was the predominant risk factor of LEA, accounting for 64.61% of the patients. This percentage of DM has increased compared to 48.2% previously found at SOTH during 2000–2004 [20]. Patients with undertreated or not monitored DM, who often discover their disease with the limb problem stirring LEA, frequently undergo LEA complications; as found in previous studies done in Togo [21, 35].

The diabetic patients in our series had a significantly high hyperleukocytosis or leukopenia. However, there was no significant difference in infection rates between patients with and without DM in the early postoperative complications of LEA. In our setting, the first goal for patients with DM was to remove the gangrene and control infection. Therefore, the indication was major amputation, most in the leg, far from the foot infection. In contrast, many recent studies performed in Western countries reported a very low incidence of below-knee amputations (25.8–9.5%) and an increased number of foot amputations due to the high rate of revascularisation and endovascular surgeries [38]. In our setting, there were no revascularization surgeries, patients presented late, and, most of all, with a long duration from the indication of LEA to the surgery. The patients used to attempt to salvage the limb by all means, returning home for indigenous treatment before returning hospital for the LEA. In these conditions, the below-knee amputation was still the best option and should be preferable to serial amputations, overambitious desperate attempts to foot salvage [32, 33]. The CCI was below 2 in 89.19%, and the mortality rate was 7.14%; thus, the CCI also sustained the statement about the severity of the patient's disease, showing that the deaths are not directly related to the comorbidities or other health conditions the patients may have had [26, 27].

Our data missed post-histologic analysis of the amputated limb to reveal possible tumours. This deficiency could be due to a religious tradition, based on which, in case of death, patients’ families can request the amputated limbs for burial alone or with the whole patient’s body [39–41].

In our study, the mean duration of hospitalization was 36.3 days. In developing countries, the mean hospital stay for LEA was more than 2 weeks and longer than that in developed countries. The hospital admission time of the patients with LEA due to trauma was significantly longer than others. This long hospitalization time can be due to staged procedures in traumatic patients, from damage control surgery to the stump refinement [13, 14, 20, 33, 42].

The current pattern of LEA in Togo urges a multidisciplinary approach to improve soft tissue management after trauma, revascularization equipment, and post-surgery physiotherapy. It has also underscored the importance of a collaboration between in-hospital specialists and territory stakeholders. The involved specialists are orthopaedists, general or plastic or vascular surgeons, endocrinologists, cardiologists, geriatricians, internal medicine specialists, nutritionists, psychiatrists, prosthetics confectioners, physiotherapists and the territory stakeholders can be general practitioners and patients’ associations. This collaboration should focus on the lower limb pathologies leading to LEA early detection, and educational campaigns to prevent chronic cardiovascular diseases, especially DM.

Our findings have prompted the importance of organizing a structured surveillance system as earlier recommended by Abbas et al. [19] that considers the socio-economic context for comprehensive screening and the follow-up costs in charge of the patients.

Future studies about LEA in Togo should be prospective, focused on the profile of the subset of the patients with DM undergoing this disabling surgery, and leverage a specific database.

## Conclusion

Notwithstanding an overall decreased incidence of LEAs at SOTH in Togo during 2010–2020 compared with previous decades, the still remarkable incidence in most cases correlates to chronic cardiovascular and cardiometabolic diseases, especially DM. This pattern of LEA imposes a multidisciplinary approach for screening programs, databases, a specific surveillance system, and clinical protocols and imaging monitoring.

Patients who must undergo LEA in Togo should benefit from an improved surgical approach and post-surgery physiotherapy. Moreover, sensitizing all the healthcare stakeholders and the population through dedicated educational campaigns for DM prevention can reduce the high rate of LEA.

## Data Availability

The datasets generated and/or analysed during the current study are available from the corresponding author on reasonable request.

## Abbreviations

AKA: Above knee amputation
BKA: Below knee amputation
CCI: Charlson comorbidity index
CDC: Centers for Disease Control and Prevention
CI: Confidence interval
DM: Diabetes mellitus
F: Fisher test
Fig.: Figure
LEA: Lower extremity amputation
Nig.: Nigeria
OR: Odds ratio
*P*: Cell value
SD: Standard deviation
STROBE: Strengthening the reporting of observational studies in epidemiology
SOTH: Sylvanus Olympio Teaching Hospital
US: United States
χ^2^: Chi 2 test
